# Cook Co. Hospital

**Published:** 1865-12

**Authors:** 


### EDITORIAL AND MISCELLANEOUS.

### COOK COUNTY HOSPITAL.

The Chicago City Hospital was taken possession of by the National government during the war, and used as an Opthalmic Hospital. The close of the war rendering it no longer necessary for this purpose, it was restored to the city government, who have transferred it to the County, and it is to be opened as a county institution under the name of the Cook County Hospital. It is a large and commodious building, and well arranged for the purposes for which it was designed, and being the only public Hospital in a county numbering more than 200,000 people, gives assurance that it will be filled with every variety of disease. The Medical and Surgical Board is composed of well known members of the profession in this city, and their appointment guarantees to the inmates of the Hospital skillful and faithful attendance. The Board is as follows :

Attending Surgeons.—George K. Amerman, M. D.; R. Bogue, M. D.; Chas. G. Smith, M. D.

Consulting Surgeons.—Jos. W. Freer, M. D.; Wm. Wagner, M. D.

Attending Physicians.—Thos. Bevan, M. D.; J. P. Ross, M. D.; H. W. Jones, M. D.

Consulting Physicians.—H. A. Johnson, M. D.; R. C. Hamill, M. D.

Pathologist.—Henry M. Lyman, M. D.

Warden.—Benjamin Chase.

Matron.—Mrs. B. Chase.

